# Machine Learning Algorithms Identify Pathogen-Specific Biomarkers of Clinical and Metabolomic Characteristics in Septic Patients with Bacterial Infections

**DOI:** 10.1155/2020/6950576

**Published:** 2020-07-27

**Authors:** Lingling Zheng, Fangqin Lin, Changxi Zhu, Guangjian Liu, Xiaohui Wu, Zhiyuan Wu, Jianbin Zheng, Huimin Xia, Yi Cai, Huiying Liang

**Affiliations:** ^1^Guangzhou Women and Children's Medical Center, Guangzhou Medical University, Guangzhou, China; ^2^School of Software Engineering, South China University of Technology, Guangzhou, China; ^3^Pediatric Intensive Care Units, Guangzhou Women and Children's Medical Center, Guangzhou Medical University, Guangzhou, China; ^4^Department of Pediatric Surgery, Guangzhou Women and Children's Medical Center, Guangzhou Medical University, Guangzhou, China

## Abstract

Sepsis is a high-mortality disease that is infected by bacteria, but pathogens in individual patients are difficult to diagnosis. Metabolomic changes triggered by microbial activity provide us with the possibility of accurately identifying infection. We adopted machine learning methods for training different classifiers with a clinical-metabolomic database from sepsis cases to identify the pathogen of sepsis. Records of clinical indicators and concentration of metabolites were obtained for each patient upon their arrival at the hospital. Machine learning algorithms were used in 100 patients with clear infection and corresponding 29 controls to select specific biosignatures to discriminate microorganism in septic patients. The sensitivity, specificity, and AUC value of clinical and metabolomic characteristics in predicting diagnostic outcomes were determined at admission. Our analyses demonstrate that the biosignatures selected by machine learning algorithms could have diagnostic value on the identification of infected patients and Gram-positive from Gram-negative; related AUC values were 0.94 ± 0.054 and 0.80 ± 0.085, respectively. Pathway and blood disease enrichment analyses of clinical and metabolomic biomarkers among infected patients showed that sepsis disease was accompanied by abnormal nitrogen metabolism, cell respiratory disorder, and renal or intestinal failure. The panel of selected clinical and metabolomic characteristics might be powerful biomarkers to discriminate patients with sepsis.

## 1. Introduction

Despite advances in the management of septic patients, sepsis remains the most common cause of death in noncardiac intensive care units (ICUs) and is the 10th leading cause of death [1]. Early and appropriate use of antibiotics is a key component for reducing mortality of affected patients with severe sepsis and septic shock [2]. However, initial use of antibiotics is usually empirical because accurate and reliable rapid diagnostic methods to identify specific pathogens are not currently available [3]. Conventional experiments, including blood culture and biochemical identification, immunological assay, nucleotide probe hybridization, and PCR amplification, share a common shortcoming: only few kinds of bacteria can be identified in a long-term cycle of experiment [4]. These serial procedures are hard to use for rapid and simultaneous detection of multiple pathogenic bacteria. Therefore, rapid and accurate prediction of microbial etiology remains a continuing challenge for clinicians and medical workers worldwide [5].

Recent advances—in particular, the development of systems biology tools such as metabolomics—have enabled key insights into the change of chemical environment in sepsis [6]. In addition, their use to explore the difference among different bacterial pathogens is critical for the development of improved experiments to achieve rapid and parallel identification of many common pathogenic bacteria in one experiment. For example, in vitro and in vivo studies have revealed fundamental differences in host response to infection, including an increase in glycolytic intermediates with decreased flux through the TCA cycle and elevated multiple inflammatory markers [7]. Different infectious mouse models showed that *Streptococcus pneumoniae* and *Staphylococcus aureus* pneumonia induce distinct metabolic responses [8]. Continuous broadening of the applications of plasma metabolomic biosignatures in prediction of infectious disease progression is evident from a surge of publications in this field, including virus infection [9], bacterial infection [10], protist infection [11], fungal infection [12], and parasite infection [13, 14]. All research studies suggest that through exploring plasma pathogen-specific metabolomic biosignatures, we may develop a method allowing fast and reliable microorganism identification of sepsis cases. By comparison, however, metabolomics in microorganism discrimination is a relatively late comer and no attempts have been undertaken to extensively investigate the value of metabolomic biosignatures in direct identification of microbial etiology among sepsis patients.

The limitation of metabolomic datasets from high-throughput technologies lies in the small number of samples versus the larger number of features represented. Machine learning methods can help integrate these large-scale omics datasets and identify key features from the dataset. Particularly, machine learning and systems metabolomic approaches can integrate clinical day and metabolite data by using data mining and predictive algorithms, pointing out that the approaches can support a more powerful identification of pathogens than an analysis using only a single data type [15, 16]. Therefore, there is an acute need for the development of the microorganism identification platform based on the framework of machine learning and metabolomic approaches using clinical data and metabolite data.

Thus, to shorten the time for identification of microorganism of sepsis patients, in the present study, we adopted machine learning methods for training different classifiers with a clinical-metabolomic database from sepsis cases to identify the pathogen of sepsis.

## 2. Materials and Methods

### 2.1. Data Sources

The data came from the Community Acquired Pneumonia and Sepsis Outcome Diagnostics (CAPSOD) study (ClinicalTrials.gov NCT00258869). The patient samples and clinical relative measurements have been described in detail previously by Langley et al. [17]. Briefly, 1152 patients with suspected sepsis (≥2 systemic inflammatory response syndrome (SIRS) criteria and certain infection) from emergency departments at three hospitals of the United States between 2005 and 2009 were enrolled into the CAPSOD cohort. 129 subjects were chosen in this retrospective study: 100 patients with clear infection (Gram-positive (*N* = 67) and Gram-negative organisms (*N* = 33)) and corresponding 29 controls. Besides, three common pathogenic bacteria that caused sepsis, including *Staphylococcus aureus* (*N* = 27), *Streptococcus pneumoniae* (*N* = 28), and *Escherichia coli* (*N* = 17), were also chosen for classification. Patient demographics, medical history, physical test, and acute illness scores (APACHE II) were recorded; corresponding blood samples with blood routine examination were collected at admission (*t*_0_). Patients were clearly defined as culture-negative or as confirmed bacterial infections by Gram-positive, Gram-negative organisms, and others by the microbiological analysis of cultures or PCRs.

### 2.2. Metabolomic Biomarkers

Metabolites in plasma with a mass-to-charge (*m*/*z*) ratio of 99-1000 were measured using ultra-high-performance liquid chromatography-tandem mass spectrometry (UPLC-MS/MS; positive or negative mode) and gas chromatography-mass spectrometry (GC-MS). The number of metabolites with clearly annotated information was 214 at *t*_0_, which can be grouped as follows: (1) amino acid, (2) carbohydrate, (3) cofactors and vitamins, (4) energy, (5) lipid, (6) nucleotide, (7) peptide, and (8) xenobiotics. We excluded xenobiotics from our study, in consideration of confusion of unusual substrates containing drugs or harmful food additives.

### 2.3. Data Analyses

According to the purpose of research, we separated the samples at *t*_0_ or *t*_24_ into two predictive subsets: subset 1: validation of infection or not; subset 2: distinction between Gram-positive and Gram-negative. Categorical variables of each group were presented as percentages, and continuous variables were showed as mean ± standard deviation. Mann-Whitney tests were applied to evaluate the relationship between two patient groups. The level of statistical significance for all analyses was *p* < 0.05. We used XGBoost [18] combined with three feature selection methods: variance threshold [19], maximal information coefficient (MIC) [20], and relief [21], to perform classification selection among aforementioned 6 predictive subsets, respectively, using the total variables obtained from clinical and metabolomic characteristics. The processing of feature selection can be summarized as three steps. Firstly, the most informative individual features were chosen. Then, feature subsets ranged from one to maximum sizes were tested for optimum prediction effect by 10-fold crossvalidation. Lastly, voting was applied for selecting the features with the major amount of votes. Voting is a combined strategy for classification in machine learning. The basic idea is to select the output with the most class by machine learning algorithms. The model's performance was estimated by test set, which was measured on sensitivity, specificity, and AUC. To reduce overfitting and derive a reliable estimate of the performance of the model, this process was repeated 500 times with random training and testing sets. The final performance of each model was obtained by averaging over the 500 evaluations. Before applying machine learning algorithms, missing values were interpolated by adopting multivariate imputation using the *DMwR* R package [22], which imputed an incomplete variable by generating the corresponding values among the set of adjacent samples. The standard normalization with mean of 0 and variance of 1 was performed for the features to reduce the effect of large feature range variation. The version of *R* software used for analysis in this article was 3.4.0. The *sklearn* and *numpy* libraries of python, version 3.5.0 [23], were adopted for the implementation of machine learning models.

### 2.4. Bioinformatics Analysis

Pathway and enrichment analyses for metabolites were performed with the MetaboAnalyst, which is a comprehensive tool for metabolomic analysis and interpretation [24] (http://www.metaboanalyst.ca/). All of the metabolites selected by the model in the test datasets were feed into the MetaboAnalyst pathway analysis program. After processing and normalization of metabolite components, the pathway analysis algorithm of the hypergeometric test was applied to search statistically significant pathways, which was defined as *p* value < 0.05. Similarly, the features of efficiently distinguishing infection were performed using metabolite set enrichment analysis (MSEA) among 344 blood disease-associated metabolite sets to explore metabolic diseases associated with sepsis.

## 3. Results

### 3.1. Characteristics of Clinical and Metabolomic Biomarkers for Patients Infected by Bacteria

A total of 100 microbiologically well-defined patients were enrolled in this study, together with 29 noninfection controls. Of these patients, 67 (67%) were diagnosed with Gram-positive infection (Table 1).

**Figure 1:**
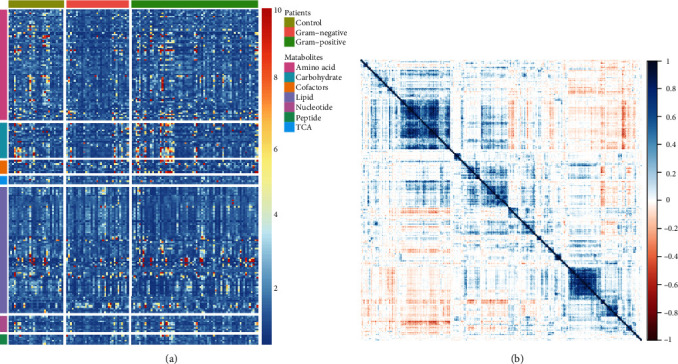
Characteristics of metabolomic biomarkers for septic patients at hospital admission. (a) The heat map showed that 199 metabolomic biomarkers can be categorized into 7 kinds of metabolites among patients (Gram-negative or Gram-positive) and controls. (b) Correlation analysis of 199 metabolomic biomarkers illustrated the 3 clearest clusters.

A broad range of clinical and metabolomic biomarkers were measured at hospital admission and 24 hours later. 199 metabolomic biomarkers were chosen for following feature selection and model prediction at *t*_0_, which covered the tricarboxylic acid (TCA) cycle, protein metabolism, and lipid transformation (Figure 1(a)). Hierarchical clustering of pairwise Pearson correlations of 199 metabolomic biomarkers in 129 subjects at *t*_0_ illustrated the 3 clearest clusters (Figure 1(b)).

### 3.2. Feature Selection Methods Identify Patients with Sepsis

**Figure 2:**
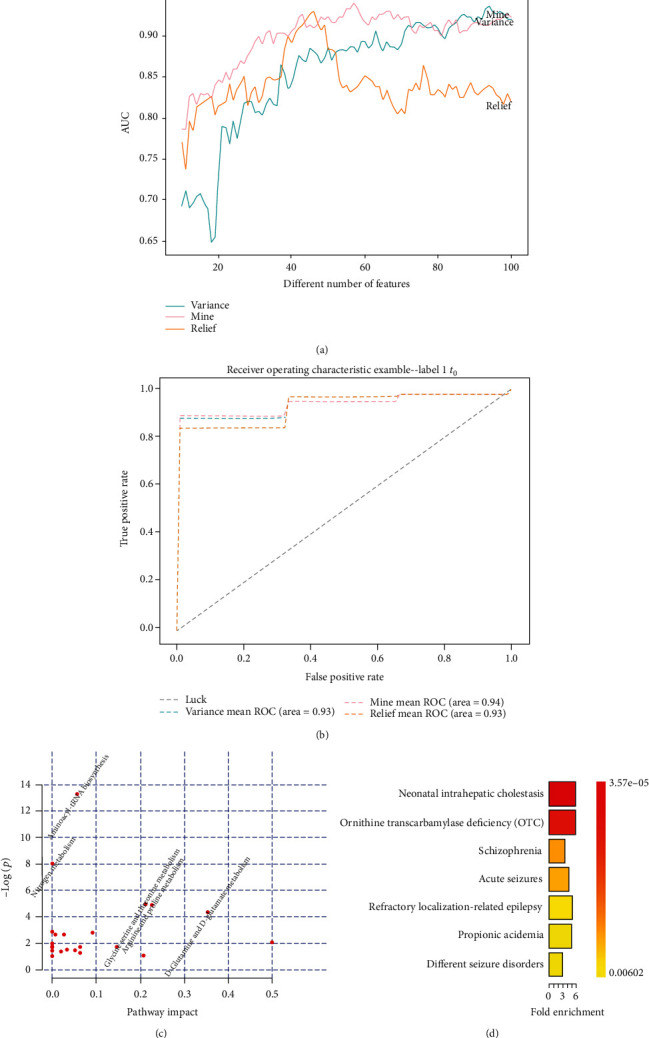
Identification of clinical and metabolomic features associated with sepsis at hospital admission. (a) Performance of feature selection models based on variance threshold, MIC, and relief for the prediction of sepsis against all other controls. Image illustrated area under the receiver operating characteristic curve (AUC) values change depending on the number of features. (b) Sensitivity and specificity of the selected features by receiver operating characteristic analysis. (c) Pathway analysis of the metabolites filtered by the MIC model. (d) Enrichment analysis of blood disease-associated metabolites.

Initially, we attempted to identify patients with sepsis from noninfected controls defined as systemic inflammatory response syndrome (SIRS). The prediction model trained on training sets using XGBoost classifier was applied on testing sets, and the model performance was evaluated according to the area under the receiver operating characteristic curve (AUC). From the head of the ranked list of variables, new models were built and assessed on feature subsets by adding one variable at a time in 10-fold crossvalidation (Figure 2(a)). In consideration of divergence among each training set leading to inconsistency of selected features, we adopted a voting method to achieve optimal features with the major amount of votes (Supplementary Excel file: S table 1A). The final feature list based on MIC was reassessed on the testing sets to successfully demonstrate availability of discrimination between septic infections and SIRS, with the optimum biomarker combination comprising 57 features (AUC = 0.94 ± 0.054, sensitivity = 0.99 ± 0.019, and specificity = 0.53 ± 0.165). In comparison, the predictive performance of features determined by variance threshold and relief was slightly less powerful (AUC = 0.94 ± 0.038 or 0.93 ± 0.044), indicating lower degrees of sensitivity (sensitivity = 0.97 ± 0.040 or 0.95 ± 0.042) and specificity (specificity = 0.38 ± 0.147 or 0.30 ± 0.216) and comprising 94 and 46 features, respectively (Figure 2(b), Supplementary Excel file: S table 1B). Although the 3 feature selection models yielded different sets of biomarkers, clinical variables like platelet count, white cell count, and blood lactate were selected in each, which suggested that the traditional indicators showed an auxiliary effect on the discrimination of septic infections.

Besides, we performed pathway analysis of the metabolites filtered by the MIC model. Five pathways, like aminoacyl-tRNA biosynthesis; nitrogen metabolism; glycine, serine, and threonine metabolism; arginine and proline metabolism; and D-glutamine and D-glutamate metabolism, were identified with significant *p* values (*p* < 0.05) (Figure 2(c), Table 2). Interestingly, after enrichment analysis of disease-associated metabolite sets, the metabolites were highly enriched for disease categories relating to neonatal intrahepatic cholestasis, ornithine transcarbamylase deficiency, schizophrenia, acute seizures, refractory localization-related epilepsy, propionic acidemia, and different seizure disorders (*p* < 0.05), which implied deterioration of liver metabolism in septic patients (Figure 2(d), Supplementary Excel file: S table 1D).

In contrast to conventional statistical methods focused on significant difference of variables, the features selected by MIC were not all pronouncedly different between patients with sepsis and patients with SIRS (Supplementary Excel file: S table 1E). Our analyses demonstrated the advantages of feature selection approaches to successfully separate two types of patients. Overall, the method of feature selection can effectively identify septic patients from SIRS by combining clinical and metabolomic biomarkers.

### 3.3. Biomarkers for Effective Discrimination between Gram-Negative and Gram-Positive Bacteria

**Figure 3:**
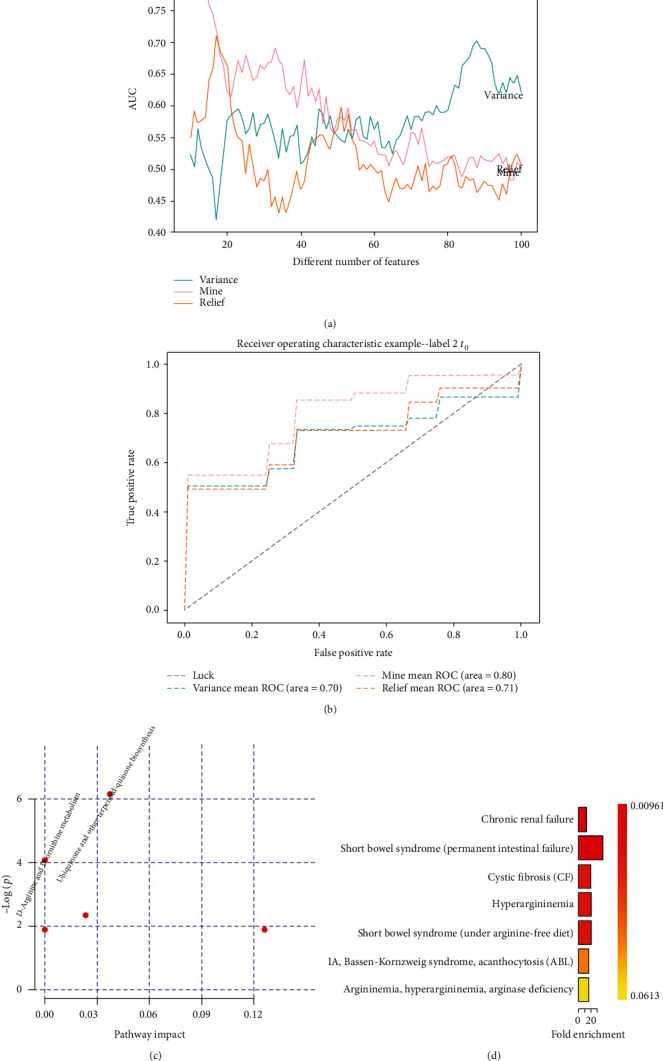
Clinical and metabolomic features for effective discrimination between Gram-negative and Gram-positive bacteria at hospital admission. (a) Performance of feature selection models based on variance threshold, MIC, and relief for the prediction of sepsis against all other controls. Image illustrated area under the receiver operating characteristic curve (AUC) values change depending on the number of features. (b) Sensitivity and specificity of the selected features by receiver operating characteristic analysis. (c) Pathway analysis of the metabolites filtered by the MIC model. (d) Enrichment analysis of blood disease-associated metabolites.

It is a challenge to identify Gram-negative from Gram-positive infection for new hospitalized patients in the clinic. Meanwhile, machine learning offers a way to solve this dilemma. Here, the MIC model was employed to select biomarker signatures that reliably distinguished patients with Gram-negative from samples with Gram-positive bacterial infection (Figure 3(a) and Supplementary Excel file: S table 2A). According to the filtered 10 features voted by 10-fold crossvalidation, the classifier showed great potential in identifying between Gram-negative and Gram-positive (AUC = 0.80 ± 0.085, sensitivity = 0.86 ± 0.063, and specificity = 0.48 ± 0.171) (Figure 3(b), Supplementary Excel file: S table 2B).

Pathway analysis pointed out divergency of ubiquinone and other terpenoid-quinone biosynthesis and D-arginine and D-ornithine metabolism (Figure 3(c), Supplementary Excel file: S table 2C). Chronic renal failure and short bowel syndrome (permanent intestinal failure) were regarded as major metabolic diseases adopting enrichment analysis of disease-associated metabolite sets (Figure 3(d), Supplementary Excel file: S table 2D).

## 4. Discussion

Due to the time-consuming identification of pathogens and rapid progression of symptoms, patients with sepsis are at high risk of insufficient quality of treatment and poor postdischarge outcomes [25]. Early diagnostic strategies for sepsis are thus urgently demanded to improve patient survival rate by reducing the time required to establish the diagnosis and provide appropriate treatment to avoid unnecessary antibiotics. In this study, machine learning algorithms were utilized to create a prediction system for accurate discrimination of patients with sepsis through combination of clinical and metabolomic biomarkers. Depending on the feature sets selected by machine learning models, classifiers achieved satisfactory performance to distinguish (1) patients with sepsis from SIRS and (2) Gram-negative infection from Gram-positive, with AUC of 0.94 ± 0.054 and 0.80 ± 0.085, respectively. Pathway analysis pointed out that these feature sets were mainly involved in the following metabolic processes: aminoacyl-tRNA biosynthesis; nitrogen metabolism; ubiquinone and other terpenoid-quinone biosynthesis; primary bile acid biosynthesis; glycine, serine, and threonine metabolism; alanine, aspartate, and glutamate metabolism; and glycerophospholipid metabolism. Moreover, the break of concentration balance of these feature sets exacerbated metabolic disorder of the liver, intestines, and circulatory system.

It is different from previous studies which only explored several metabolites to identify bacterial infection in septic patients or just screened out the metabolites that are significant between the infective group and controls [26] [27] [28]. Traditional strategies using the significant difference of metabolites are unable to achieve accuracy of discrimination of infectious agents [26]. Along with medical data explosion, machine learning has gained unprecedented advantages among disease diagnosis, outcome prediction, and medication instruction, especially identification of pathogens via combination of demographic data and multiple dimension omics data [15, 16]. Here, we applied the supervised machine learning model to discriminate bacterial infections [29]. And we applied feature selection methods to provide best feature combination to precisely diagnose pathogens. Variance threshold is a simple baseline approach to remove features which cannot pass the defined threshold [19]. Compared to mutual information which is a measure of strength of the linear or nonlinear association between two variables, Maximal Information Coefficient (MIC) converts mutual information into a metric by searching for an optimal discretization method to overcome the difficulties of normalization and discretization. The relief algorithm gives different weights to features according to the correlation of each feature and category; the feature whose weight is less than a threshold will be removed [30]. Our conclusion showed that machine learning not only improved the accuracy of bacterial infection prediction but also provided the cognition about the metabolic process of organisms in the process of sepsis by the features selected with three models.

Although the 3 feature selection models yielded different sets of biomarkers, clinical variables like platelet count, white cell count, and blood lactate were selected in each, which suggested that the traditional indicators showed an auxiliary effect on the discrimination of septic infections [31]. As traditional clinical indicators, like platelet count, white cell count, or blood lactate, all of them are considered valuable biomarkers to monitor the progress of sepsis. The normal range of platelet count in adults is 150000–400000/*μ*l. On the contrary, this value fell to a lower level of 80000/*μ*l in most severe septic patients [32]. Therefore, platelet count is an important signal in the diagnosis of sepsis, which suggests that greater levels of platelet count are associated with higher risk of death. White cell count is very sensitive to pathogens, which plays a key role to determine sepsis with too low or high levels [27]. Mitochondrial damage induced by hypoperfusion due to infection can inhibit aerobic respiration and promote lactate production [32]. Although these clinical characteristics can be used as a detection index on sepsis, the sensitivity and specificity of AUC are not satisfactory.

Metabolomic profiling reflects flux of metabolic substrates from both the host and the pathogen in vivo, which offers direct insights into the chemical environment for infectious diseases [33]. Early research pointed out that during the sepsis processing in rat, protein decomposition promoted the increase of nitrogen in the blood and accelerated the metabolism of nitrogen [28]. Karinch et al. also demonstrated that severe infection causes the release of glutamine from skeletal muscle and accelerated absorption by the liver [21]. Hence, the metabolic response to sepsis induces changes of protein and amino acid metabolism, which increase protein degradation, amino acid decomposition, and nitrogen metabolism. Interestingly, in our study, we also selected blood metabolites involved in the identification of pathogens by the machine learning method. Severe sepsis-induced multiorgan failure is accompanied with mitochondrial dysfunction [34]. Coenzyme Q10 (CoQ10) is regarded as a key cofactor in the mitochondrial respiratory chain, whose levels were associated with the processing of septic shock [35]. Even more, researchers tried to inject ubiquinol (reduced CoQ10) into septic shock patients and tested curative effect [36]. In our prediction model, the pathway of ubiquinol was filtered by the feature selection model to identify Gram-negative from Gram-positive *Escherichia coli* infections among common bacteria causing sepsis. Recently, high-performance liquid chromatography-high-resolution mass spectrometry (HPLC-HRMS) revealed that serum bile acid concentration was significantly fluctuation in both septic adults and neonates compared to healthy controls [37]. The cause of the phenomenon is endotoxin, produced by microbial activity, interfering the normal signaling pathways, greatly reducing bile flow, and resulting in sepsis-associated cholestasis [38].

Besides amino acid or bile acid metabolism, glycerophospholipid levels were also proved to be a kind of great biomarkers for developing prognostic tools [39]. Compared to the SIRS level, sepsis samples showed significantly higher glycerophospholipid concentration due to response to infection-induced inflammation [40]. Findings demonstrated a downregulation of lipoproteins in the circulatory system, in particular high-density lipoprotein and low-density lipoprotein, which promoted the increase of glycerophospholipid [41, 42]. Parts of enzymes are located in the inner membrane of mitochondria, while impaired mitochondrial function due to tissue hypoperfusion in sepsis suppresses function of enzymes for lipid catabolism, which further intensifies the increase of glycerophospholipid concentration [43].

Multiple organ failure induced by sepsis, such as the liver, kidneys, and intestines, is a hallmark of sepsis [44]. We found that certain metabolic pathways of bacterial infection coincide with the metabolic pathways of genetic diseases, like Lesch-Nyhan syndrome. It is a rare inherited disorder caused by a deficiency of the enzyme hypoxanthine-guanine phosphoribosyltransferase (HGPRT), which promotes the accumulation of uric acid in the child body. Growing evidence demonstrated the relationship between the levels of serum uric acid and septic diagnosis or prognosis [45, 46]. Similarly, the most common form of ornithine transcarbamylase deficiency is often accompanied by dysfunction of the urea cycle. Certain reports showed that hyperammonemia from the accumulation of urea generally appears in patients with severe septic shock [47]. In addition, features applied to discrimination of Gram-negative from Gram-positive were enriched in chronic renal failure and short bowel syndrome. Severe sepsis or septic shock will cause kidney failure, which is often among the first to be affected. Based on observational clinical investigation, kidney failure will accelerate mortality, caused by uremia or microelement reabsorption disorder [48]. Abnormal intestinal function or biliary atresia also induces higher risk for infectious complications.

Despite of excellently discriminatory results, some important limitations of our predictive system should be noticed. The data including clinical and metabolomic characteristics applied for feature selection or model prediction were derived from the article published by Langley et al. The system development and panel evaluation were only based on above data; additional validation with more patients should be tested before medical practice. Furthermore, the overlap rate of biomarkers was low among panels, which increased the cost and time of detection. It would be useful to explore more efficient machine learning methods and optimize panel biomarker candidates.

## 5. Conclusion

In conclusion, it is very meaningful to successfully develop an efficient classifier utilizing the combination data of clinical and metabolomic features to identify specific pathogens in septic patients. The approach combining machine learning and specific biosignatures provides an efficient diagnosis strategy among septic patients with low cost and time-saving, which is only based on several clinical and metabolite indicators. According to the given diagnosis purposes, the biomarkers generated by each label can be developed into corresponding diagnosis kits. The panel containing specific biomarkers will speed up the detection efficiency of bacterial infection. Without doubt, these panels could improve the accuracy of diagnosis and reduce the mortality of the septic patients.

## Figures and Tables

**Table 1:** Characteristics of the patients in our study.

Clinical variable	Control		Gram-positive		Gram-negative	
	No.	%	No.	%	No.	%
*n*	29	—	67	67	33	33
Age (years)	65.79 ± 13.40		56.33 ± 17.04		60.18 ± 17.80	
<50	4	13.79	23	34.33	8	24.24
50-60	8	27.59	17	25.37	8	24.24
60-70	7	24.14	12	17.91	8	24.24
70-80	4	13.79	7	10.45	4	12.12
≥80	6	20.69	8	11.94	5	15.15
Sex						
Male	12	41.38	41	61.19	17	51.52
Female	17	58.62	26	38.81	16	48.48
Race						
Black	21	72.41	45	67.16	21	63.64
White	7	24.14	17	25.37	11	33.33
Other	1	3.45	5	7.46	1	3.03
Pathogen						
*S*. *aureus*	N/A		27		N/A	
*S*. *pneumoniae*	N/A		28		N/A	
*E*. *coli*	N/A		N/A		17	
APACHE II	16.26 ± 7.43		15.55 ± 7.18		16.79 ± 8.20	
Temperature (°C)	36.82 ± 1.08		38.01 ± 1.44		38.05 ± 2.07	
MAP (mmHg)	89.31 ± 29.58		79.06 ± 17.94		77.12 ± 16.09	
Heart rate	107.07 ± 19.71		119.61 ± 21.24		108.03 ± 22.54	
Respiratory rate	25.24 ± 6.32		26.61 ± 8.30		23.22 ± 6.57	
Serum sodium (mM)	136.86 ± 4.33		136.64 ± 6.03		136.52 ± 4.05	
Serum potassium (mM)	4.69 ± 1.26		4.30 ± 1.10		4.19 ± 0.98	
Serum creatinine (mg/dl)	2.71 ± 3.67		3.34 ± 3.88		2.45 ± 2.48	
Blood lactate (mg/dl)	4.29 ± 4.38		2.75 ± 2.14		3.30 ± 2.82	
Hematocrit	34.79 ± 6.45		36.30 ± 6.73		34.82 ± 6.43	
White cell count	10.80 ± 4.13		17.16 ± 18.96		16.27 ± 9.42	
Platelet count	275.52 ± 97.19		226.19 ± 135.68		243.03 ± 110.35	

Data are presented as mean ± standard deviation. MAP: mean arterial pressure; N/A: not available.

**Table 2:** Pathway analysis of the metabolites associated with sepsis at hospital admission.

	*p* values	Pathway impact
Aminoacyl-tRNA biosynthesis	1.70*E*-06	0.055
Nitrogen metabolism	0.00034	0
Glycine, serine, and threonine metabolism	0.0068	0.21
Arginine and proline metabolism	0.0072	0.22
D-Glutamine and D-glutamate metabolism	0.013	0.35

## Data Availability

The data used to support the findings of this study are available from the corresponding authors upon request.

## Supplementary Materials

Supplementary Materials S2 Table A: selected features and corresponding weights associated with sepsis at hospital admission. S4 Table A: selected features and corresponding weights for effective discrimination between Gram-negative and Gram-positive bacteria at hospital admission.
